# Focal Cerebral Ischemia Induces Global Subacute Changes in the Number of Neuroblasts and Neurons and the Angiogenic Factor Density in Mice

**DOI:** 10.3390/medicina59122168

**Published:** 2023-12-14

**Authors:** Vladimirs Pilipenko, Zane Dzirkale, Rebeka Rozkalne, Jolanta Upite, Farida Hellal, Nikolaus Plesnila, Baiba Jansone

**Affiliations:** 1 Department of Pharmacology, Faculty of Medicine, University of Latvia, Raina blvd. 19, LV-1586 Riga, Latvia; zane.dzirkale@lu.lv (Z.D.); jolanta.upite@lu.lv (J.U.); 2 Institute for Stroke and Dementia Research, University Hospital, Ludwig Maximilian University Munich, 81377 München, Germany; farida.hellal@helmholtz-muenchen.de (F.H.); nikolaus.plesnila@med.uni-muenchen.de (N.P.)

**Keywords:** angiogenesis, neurogenesis, focal ischemia, animal model, stroke recovery

## Abstract

*Background and Objectives*: Dissecting the complex pathological cascade of an ischemic stroke in preclinical models is highly warranted to understand the course of this disease in humans. Neurogenesis and angiogenesis are integral for post-stroke recovery, yet it is not clear how these processes are altered months after an ischemic stroke. In this study, we investigated the changes that take place subacutely after focal cerebral ischemia in experimental adult male mice. *Materials and Methods*: Male 12-week-old C57BL/6 mice underwent a 60 min long fMCAo or sham surgery. Two months after the procedure, we examined the immunohistochemistry to assess the changes in neuroblast (DCX) and differentiated neuron (NeuN) numbers, as well as the density of the pro-angiogenic factor VEGF. *Results*: We found decreased neuroblast numbers in both brain hemispheres of the fMCAo mice: by more than 85% in the dentate gyrus and by more than 70% in the subventricular zone. No neuroblasts were found in the contralateral hemisphere of the fMCAO mice or the sham controls, but a small population was detected in the ipsilateral ischemic core of the fMCAo mice. Intriguingly, the number of differentiated neurons in the ipsilateral ischemic core was lower by 20% compared to the contralateral hemisphere. VEGF expression was diminished in both brain hemispheres of the fMCAo mice. *Conclusions*: Our current report shows that focal cerebral ischemia induces changes in neuroblast numbers and the pro-angiogenic factor VEGF in both cerebral hemispheres 2 months after an fMCAo in mice. Our data show that focal cerebral ischemia induces a long-term regenerative response in both brain hemispheres.

## 1. Introduction

Ischemic stroke is one of the most common causes of death and disability worldwide, and is responsible for more than 5.5 million human deaths each year [1]. It is caused by the obstruction of cerebral blood vessels and insufficient cerebral blood flow, resulting in cell injury and death in the lesion area. The obstruction of blood vessels rapidly leads to ATP depletion, oxygen and glucose consumption deficits, the generation of reactive oxygen species and oxidative stress, cellular membrane injury, excitotoxicity, and brain tissue infarct [2,3]. The ischemic core is formed early during an ischemic stroke and indicates brain tissue that may not be salvageable, whereas peri-infarct tissue constitutes a region close to the core that could be protected against ischemic injury. In the subacute stage, which takes place days after a stroke and continues for up to 3–6 months, neural repair and early endogenous plasticity occur. The recovery processes in the brain after a stroke involve changes in synaptic plasticity and an increase in gliogenesis, angiogenesis, neurogenesis, and axonal sprouting [4].

In the first few days following an ischemic stroke, endogenous mechanisms are activated to ensure rapid recovery by recruiting two tightly associated processes—angiogenesis and neurogenesis [5,6,7]. Neurogenesis is the formation of new neurons from neural stem cells or neuroblasts; these cells express doublecortin (DCX), a specific microtubule-associated protein, while undergoing proliferation [8]. Newly formed neurons begin to express NeuN, a marker of differentiated neurons [9]. Neurogenesis is proven to occur at two neurogenic niches: the subventricular zone (SVZ) of the lateral ventricles and the subgranular zone of the hippocampal dentate gyrus (DG) [10]. The processes of neurogenesis occur after an ischemic stroke in both human [11] and rodent [12] brains, indicating that possible therapeutic interventions could involve the enhancement of these processes to treat ischemic stroke in patients [7]. Neuroblasts migrate from the neurogenic niches towards the site of the ischemic injury, where they mature into neurons, yet only a small fraction of these neurons survive [13].

One of the main inducers of neurogenesis is tissue hypoxia, which is associated with the release of several angiogenic factors: angiopoietins, platelet-derived growth factor, basic fibroblast growth factor, and vascular endothelial growth factor (VEGF), which is the most potent proangiogenic factor [7,14]. Angiogenesis, in fact, is one of the mechanisms that increases oxygen delivery to the stroke-affected tissue. Neovascularization is a key defensive mechanism against hypoxia, as it modulates long-term neurologic recovery after an ischemic stroke [15]. Importantly, angiogenesis serves to supply hypoxic tissues with oxygen and promote neuroblast migration from the SVZ to lesioned brain regions, supported by the previous detection of migrating neuroblasts in the blood vessels of the ischemic region of the striatum [13].

Although neurogenesis and angiogenesis have been reported in the subacute stages of ischemic stroke, there are limited data on the occurrence of these processes in the non-ischemic hemisphere of the brain after stroke induction. In this study, we induced a transient experimental ischemic stroke in healthy 3-month-old male C57BL/6N mice using a 60 min fMCAo. Two months after the fMCAo, we determined how the fMCAo altered the number of neuroblasts (DCX^+^) and differentiated neurons (NeuN^+^) in the ipsilateral and contralateral neurogenic niches of the mouse brains, as well as the cerebral cortex and striatum. Moreover, we assessed the effects of the ischemic stroke on the progression of angiogenesis (VEGF) in the cortex of both brain hemispheres in the fMCAo mice and the homologous regions in the sham controls.

## 2. Materials and Methods

### 2.1. Animals

The subjects of this study were 12-week-old male C57BL/6NCrl mice (24–27 g) from Charles River Laboratories (Sulzfeld, Germany). It has been reported that a 60 min long fMCAo results in substantially higher ischemic injury in male C57Bl/6 mice when compared to female counterparts [16].

Mice were housed as groups (5–6 animals per cage) in environmentally enriched cages (dimensions: W:395 × D:346 × H:213 mm), located in individually ventilated stainless-steel racks (GR900, Tecniplast, Buguggiate, Italy) within a controlled environment (temperature of 25 ± 1 °C; humidity between 50 and 60%; and a 12 h day/night cycle with lights on from 07:00 to 19:00). Each cage contained autoclaved aspen wood chips (1031004, LBS-Biotech, Reigate, UK) in addition to enrichment items, including a polycarbonate tunnel (K3487), crawl ball (K3329), aspen blocks (1023005), and aspen wood wool (1034005) obtained from LBS-Biotech (UK). All mice had ad libitum access to filtered tap water and a standard pelleted chow (19.2% protein, 4.1% fat, 6.1% fiber, and 5.9% ash) (1324, Altromin, Mucedola, Settimo Milanese, Italy). The mice were randomized to a sham-operated group (n = 7) or an fMCAo group (n = 5).

### 2.2. Ethics Statement

The study design, surgical manipulations, post-operative care, and humane endpoints were carried out in accordance with the EU Directive 2010/63/EU, the ARRIVE guidelines, and local laws and policies on the protection of animals used for scientific purposes. Approval for the experimental procedures was obtained from the Animal Ethics Committee of the Food and Veterinary Service (Riga, Latvia) (Permit Number: 100). All efforts were made to minimize animal suffering and to reduce the total number of animals needed for the study.

### 2.3. Chemicals and Antibodies

The following chemicals were purchased from Sigma-Aldrich (St Louis, MO, USA): 4′,6-diamidino-2′-phenylindole dihydrochloride (DAPI) (D9542), bovine serum albumin (A6003), Fluoromount™ aqueous mounting medium (F4680), paraformaldehyde (PFA) (P6148), phosphate-buffered saline (PBS) (P3813), Tween*^®^*20 (P2287), and Triton X-100 (X100). Normal goat serum (NGS, 04-009-1A) was acquired from Biological Industries (Cromwell, CT, USA). The primary and secondary antibodies used in this study are summarized in Table 1.

### 2.4. Filament Middle Cerebral Artery Occlusion (fMCAo) Model

To induce cerebral ischemia in mice, a 60 min long middle cerebral artery occlusion was performed using an intraluminal filament as described in [17]. The mice were 12 weeks old and weighed 24–27 g at the time of the procedure. Analgesia was induced thirty minutes before the surgery via an intraperitoneal (i.p.) injection of carprofen (5 mg/kg) and a subcutaneous (s.c.) injection of buprenorphine (0.1 mg/kg). Anesthesia was induced via 4.5% isoflurane and maintained with 1.5% to 2% isoflurane in 0.3 L/min of O_2_ and 0.7 L/min of N_2_O, using a facemask anesthesia system (16-2025, Highland Medical, Pomona, NY, USA). During surgery, the body temperatures were maintained (37.0 ± 0.5 °C) using a feedback-controlled heating pad (DC Temperature Controller System, FHC, Bowdoin, ME, USA). Although the blood pressure was not measured, the heart rate with oxygen saturation was monitored (PhysioSuite, Kent Scientific Corporation, Torrington, CT, USA). The absence of a toe pinch response indicated deep, surgical anesthesia. Prior to the surgical incision, disinfectant was applied to the skin and the surrounding fur of the neck and the left side of the head. The blood flow in the left middle cerebral artery blood supply region was monitored using a laser-Doppler flowmetry fiber (moorVMS-LDF, Moor Instruments, Axminster, UK) placed in a small skin incision between the left eye and the left ear, on the skull bone surface above the territory of the left middle cerebral artery. After fiber placement, the mice were positioned supine, and a midline neck incision was made. The left common and left external carotid arteries were ligated and the left internal carotid artery was occluded using a microvascular clip. An fMCAo was achieved through the insertion of a silicone-coated intraluminal filament (filament size, 7-0; diameter with coating, 0.19 ± 0.01 mm; 701912PK5Re suture, Doccol, Sharon, MA, USA) through a small arteriotomy in the left common carotid artery. After filament insertion, the microvascular clip was removed from the left internal carotid artery. The left fMCAo induced a rapid drop in the blood flow in the left middle cerebral artery blood supply region to 15–20% of the pre-fMCAo baseline value. Shortly thereafter, the anesthesia was discontinued, and the mice were placed in a temperature-controlled recovery box (28.5 ± 0.5 °C) (V1200DT, MediHeat, Essex, UK). Later, the mice were re-anesthetized, and reperfusion was initiated after 60 min of ischemia through the withdrawal of the intraluminal filament. The sham surgery was performed in the same manner except that the filament was inserted and withdrawn immediately to provide equivalent irritation of the vessel wall. Two mice of the fMCAo group died after the surgery from a subarachnoid hemorrhage and were excluded from the analyses. Animals that did not show neurological deficits after the fMCAo were excluded from the study. The mortality rate of the mice after the MCAo surgery was 10%.

### 2.5. Post-Operative Care

Immediately following surgery, the mice received an s.c. injection of saline (0.5 mL) and were placed in a recovery box (2 h at 28.5 ± 0.5 °C). The mice were housed in standard cages, divided into stroke (full fMCAo procedure) and sham groups, with no more than 5 animals in one cage. The post-operative care period lasted for 1 week. A constant room temperature (24 ± 0.5 °C) was maintained in the post-operative care room. To increase the survival after the induced stroke, adequate food and water consumption was ensured for the first five days following surgery by providing the mice with soft food (powder V1530-000, ssniff-Spezialdiäten GmbH, Soest, Germany) and hydrogel (Hydrogel, ClearH2O, Westbrook, ME, USA). According to the recommendations, these were placed in Petri dishes within each cage and refilled daily [16]. A Mouse House (Tecniplast It., Buguggiate, Italy) and nesting material were placed in the cages for environmental enrichment. Post-operative analgesia was provided through an s.c. injection of buprenorphine (0.1 mg/kg) every 8–12 h for the first two days after the surgery and an i.p. injection of carprofen (5 mg/kg) every 24 h for four days after the surgery. Once daily for 7 days after the surgery, each mouse (fMCAo and sham) received an s.c. injection of a 20% glucose saline solution (0.5 mL) and Ringer’s lactate solution (0.5 mL). Animal weight was recorded before and after the fMCAo to assess recovery. The mice were continuously monitored for pain and hypothermia.

### 2.6. Brain Tissue Preparation

To determine the long-term changes induced by the fMCAo, we performed histochemical and immunohistochemical analyses. On day 60 after the fMCAo, the mice were deeply anesthetized with an i.p. mixture of ketamine/xylazine (100 mg/kg and 10 mg/kg, respectively), followed by transcardial perfusion with ice-cold saline and a whole brain extraction. Whole brains were fixed in 4% PFA overnight, then cryoprotected through submersion in 30% sucrose for 24 h. For each brain, 30 μm thick coronal sections were obtained at −26 °C ±1 °C using a cryotome (CM1850, Leica Biosystems, Richmond, IL, USA) with the coordinates from +1.18 to −2.3 mm anterior–posterior to bregma [18]. Within 200 μm, 3 sections were randomly selected for histochemical and immunohistochemical analyses.

### 2.7. Infarct Volume and Hemispheric Atrophy Detection

Coronal brain sections (30 μm thick) were cut using a freezing microtome (CM1850, Leica Biosystems, USA) and collected in 10 serial sections. Next, the sections were dehydrated with descending concentrations of alcohol, then cleared and stained with cresyl violet for 15 min. An investigator blind to the experimental groups performed the staining. The stained sections were digitized, and an image analysis was performed using ImageJ software (version 1.54f). Nissl staining was used to estimate the infarct area immediately following the fMCAo procedure and hemispheric atrophy 2 months after the fMCAo.

### 2.8. Immunofluorescence

The mouse coronal sections were rinsed in PBS containing 0.5% Tween*^®^*20 (PBS-Tw). The sections were heated in 0.01 M sodium citrate (pH = 6.0) for 20 min at 95 °C for antigen retrieval, followed by incubation for 1 h in a blocking solution (10% NGS in PBS-Tw). The sections were then incubated overnight at 4 °C in the blocking solution containing anti-VEGF (1:400) or anti-DCX (1:50). The next day, the sections were incubated for 1 h at 37 °C with AlexaFluor*^®^* 488-conjugated goat anti-rabbit IgG (1:500). The nuclei were stained with DAPI for 2 min, and then the sections were mounted, air-dried, and cover-slipped using a Fluoromount™ aqueous medium. The optical density measurements were analyzed by an investigator blinded to the experimental groups.

### 2.9. Double Immunofluorescence

Double immunofluorescence was performed to assess the co-localization of DCX+ and NeuN+ cells in the hippocampal DG. The sections were rinsed in PBS and then permeabilized in 0.4% Triton-containing PBS, followed by heating in 10 mM sodium citrate for 20 min at 95 °C and incubating for 2 h at RT in a blocking solution containing 1% BSA and 10% goat serum in PBS. The sections were then incubated overnight at 4 °C in the same blocking solution containing antibodies against both DCX (1:50) and NeuN (1:500). The next day, the sections were incubated for 2 h at 37 °C with AlexaFluor*^®^* 488-conjugated goat anti-mouse IgG (1:1000) and AlexaFluor*^®^* 594-conjugated goat anti-rabbit IgG (1:1000). The sections were then stained with DAPI, mounted, air-dried, and cover-slipped using a Fluoromount™ aqueous medium. Cell quantifications were performed as soon as possible by an investigator blinded to the experimental groups.

### 2.10. Image Acquisition and Processing

Following immunofluorescence, the mounted brain sections were digitized using the Nikon Eclipse Ti microscopy system (Nikon Europe B.V., Amstelveen, The Netherlands) equipped with 10, 20, and 40× objectives and FITC, DAPI, and TRITC filters. For the sections stained with cresyl violet (n = 3), a Pannoramic MIDI II scanner (3DHISTECH Ltd., Budapest, Hungary) with a 20× objective lens and Pannoramic Viewer 1.15.2 software were used to obtain whole brain images. The immunohistochemical and histochemical data were quantified using Fiji software (version 2.14.0), an open-source scientific image-processing software. A region of interest of equal size was selected for all the samples. The densitometry was reported as the mean intensity in arbitrary units per studied region of interest.

### 2.11. Statistical Analysis

**Figure 1:**
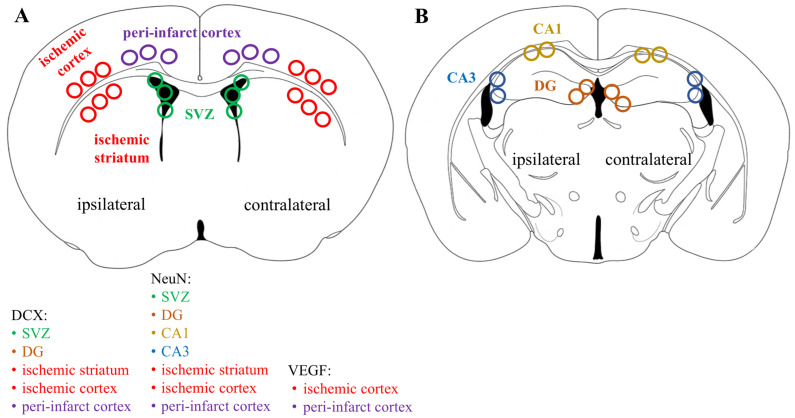
ROIs selected for immunohistochemical analyses. (**A**) ROIs in the corpus striatum slices. (**B**) ROIs in the hippocampal region slices.

The statistical analyses and graphical representations of the data were performed using GraphPad Prism (version 7.0; GraphPad Software, Inc., San Diego, CA, USA). The data were tested for normality using the Kolmogorov–Smirnov test. The data were presented as the mean ± standard deviation (SD), with *p*-values < 0.05 indicating statistically significant differences between groups. The histochemical data were analyzed using a one-tailed Student’s *t*-test. The immunofluorescence data were analyzed using a two-way ANOVA (with the hemisphere and the group as factors), followed by Holm–Sidak’s multiple comparisons test. Figure 1 shows the regions of interest (ROIs) used in the study. The ROIs were defined in the ischemia-affected cortical and striatal areas based on cresyl violet staining; these ROIs were equally mirrored to the contralateral hemisphere. Three consecutive slices per animal with a distance of 300 μm apart were selected, with the middle slice demonstrating the most pronounced ischemic lesion. The ROIs from the cerebral cortex were used for the VEGF and NeuN assessment, whereas the striatal ROIs were utilized for the detection of DCX. Homologous areas in the contralateral hemisphere and in sham-operated animals were used for quantification. Additionally, the DCX (DCX^+^)- and NeuN-positive (NeuN^+^) cell counts were performed in the neurogenic niches, namely the hippocampal DG and SVZ. The NeuN^+^ cells were also quantified in the hippocampal CA1 and CA3. The mean values of optical density and the cellular counts were analyzed between the following groups:

- Sham ipsilateral (Ipsi) vs. fMCAo ipsilateral;
- Sham contralateral (Contra) vs. fMCAo contralateral;
- Sham ipsilateral vs. sham contralateral;
- fMCAo ipsilateral vs. fMCAo contralateral.

## 3. Results

### 3.1. Generation of fMCAo Model

**Figure 2:**
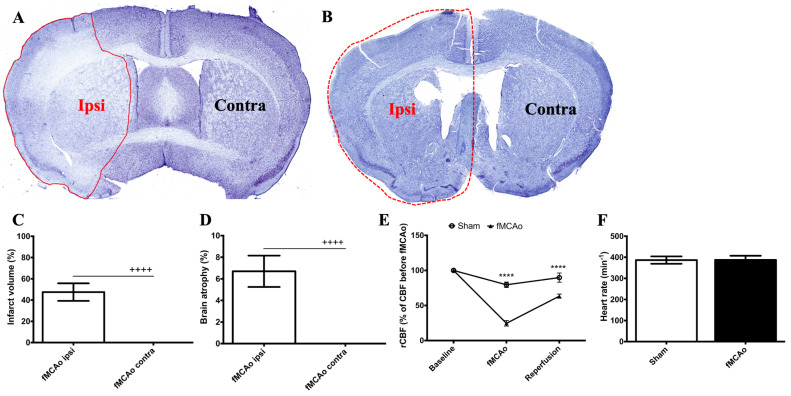
Functional parameters assessed acutely and 2 months following fMCAo. (**A**) Representative photograph depicts ischemic region 24 h after fMCAo (red line depicts the perimeter of the ischemic zone). (**B**) Representative photograph shows loss of brain hemisphere volume 2 months after fMCAo (red dotted line shows the perimeter of the contralateral hemisphere). (**C**) Graph shows the calculated infarct area as a percentage of ischemic area from the total hemisphere area (and homologous area in the contralateral hemisphere) 24 h after fMCAo. (**D**) Graph depicts the calculation of brain atrophy percentage in the ipsilateral and contralateral hemisphere (of the total hemisphere area) of fMCAo-group mice 2 months after fMCAo. (**E**) Changes in relative cerebral blood flow (rCBF) from the total cerebral blood flow (CBF). (**F**) Heart rate measurements. **** *p* < 0.0001, fMCAo vs. sham during surgery and at reperfusion; ^++++^ *p* < 0.0001, fMCAo ipsi vs. fMCAo contra.

To determine the successfulness of the fMCAo method, three animals were sacrificed 24 h after the surgery. The infarct area (evaluated with cresyl violet staining, Supplementary Figure S1) after reperfusion constituted approximately 50% of the ipsilateral hemisphere (*p* < 0.0001 vs. fMCAo contra, Figure 2A,C). According to the cresyl violet staining shown in Figure 2A, neuronal death predominantly occurred in the cerebral cortex and partially in the corpus striatum of the ipsilateral hemisphere. Two months after the induction of the fMCAo, the volume of the ipsilateral hemisphere was reduced by 7% as compared to the contralateral hemisphere, indicating significant brain atrophy (Figure 2B,D). A reduction in the rCBF was observed in both groups following the insertion of the filament, and a drop to about 25% was observed in the fMCAo group (*p* < 0.0001 compared to sham, Figure 2E). Following reperfusion, the rCBF rose to about 65% in the fMCAo group (*p* < 0.0001 compared to sham). The heart rate did not significantly differ between the groups (Figure 2F).

### 3.2. Altered Neuroblast and Neuronal Count following fMCAo

**Figure 3:**
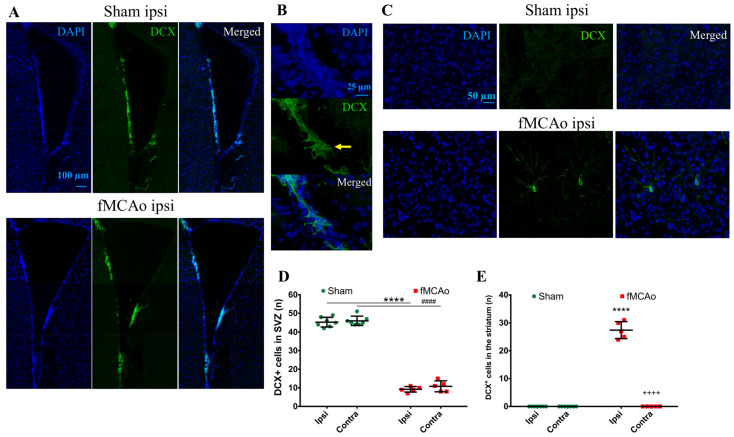
Number of DCX^+^ cells in the SVZ and striatum 2 months after fMCAo/sham surgery. (**A**) Representative images of DCX^+^ cells taken at 10× magnification. (**B**) Representative images of the SVZ region taken at 40× magnification. Yellow arrow points to DCX^+^ cells. (**C**) Representative images of striatum taken at 20× magnification. (**D**,**E**) Quantification of DCX^+^ cells in mice SVZ and striatum, respectively. Data are shown as the mean number of DCX^+^ cells ± SD (n = 7 for sham group and n = 5 for fMCAo group); differences between groups were determined using two-way ANOVA followed by Holm–Sidak’s test. **** *p* < 0.0001, fMCAo ipsi vs. sham ipsi, and ^####^ *p* < 0.0001, fMCAo contra vs. sham contra; ^++++^ *p* < 0.0001 fMCAo ipsi vs. fMCAo contra.

We first discovered a decrease in DCX^+^ cells in the SVZ of fMCAo mice compared to sham-operated animals (Figure 3A,D). Specifically, a significant reduction in DCX^+^ cells was observed in the SVZ region in the ipsilateral hemisphere of fMCAo mice (*p* < 0.0001 vs. sham ipsi) as well as in the contralateral hemisphere (*p* < 0.0001 vs. sham contra). The number of DCX^+^ cells in the SVZ of both fMCAo mouse brain hemispheres was reduced similarly, and in the sham group, these numbers also did not differ between hemispheres of the same brain. We further discovered the presence of a small population of DCX^+^ cells in the ischemic striatum of fMCAo mice (Figure 3C,E), a region neuroblasts do not originate from. We did not detect any DCX^+^ cells in the ipsilateral cortical area, in the same area in the contralateral hemisphere of the fMCAo mouse brains, or in the homologous brain areas in the sham mice.

**Figure 4:**
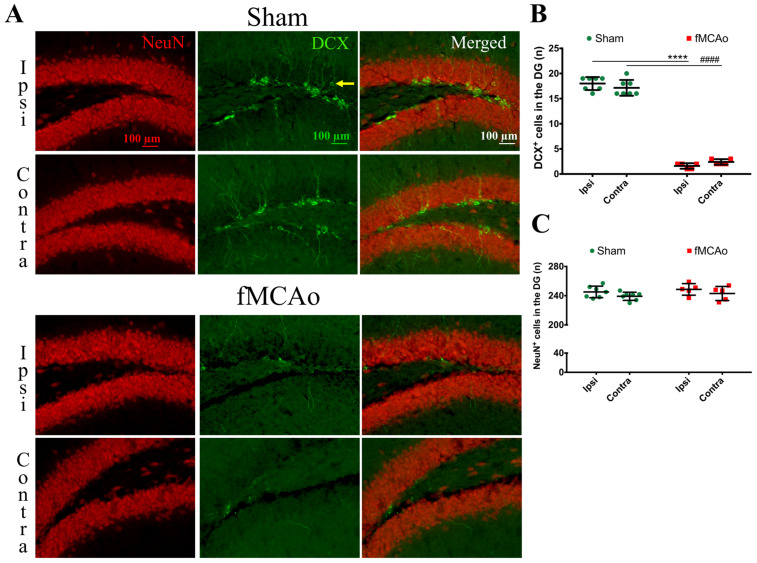
DCX^+^ and NeuN^+^ cell numbers in the hippocampal DG 2 months after fMCAo/sham surgery. (**A**) Representative images show the brain sections labeled for DCX^+^ cells (green, indicated by yellow arrow) and NeuN^+^ cells (red) at 10× magnification. (**B**) Graph depicts the quantification of DCX^+^ cells. (**C**) Graph shows the quantification of NeuN^+^ cells. Data are shown as the mean number of DCX^+^ cells ± SD (n = 7 for sham group and n = 5 for fMCAo group); differences between groups were determined using two-way ANOVA followed by Holm–Sidak’s test. **** *p* < 0.0001, fMCAo ipsi vs. sham ipsi, and ^####^ *p* < 0.0001, fMCAo contra vs. sham contra.

**Figure 5:**
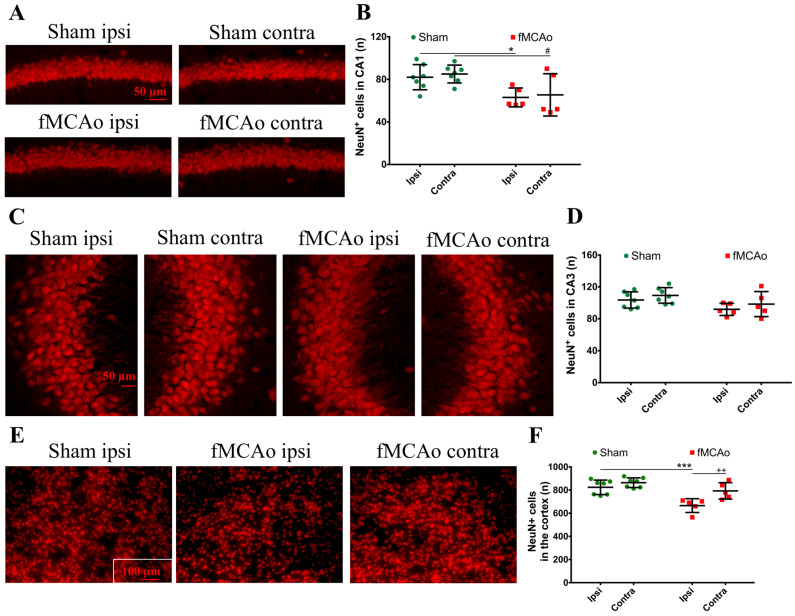
NeuN^+^ cell numbers in the hippocampal CA1 and CA3 and in the cortex of both hemispheres of fMCAo and sham-group mice. Representative photographs depict hippocampal CA1 (**A**) and CA3 (**C**) and cortical (**E**) staining of NeuN^+^ cells. Graphs depict the quantification of NeuN^+^ cells in the CA1 (**B**), CA3 (**D**), and cortex (**F**). Data are shown as the mean number of DCX^+^ cells ± SD (n = 7 for sham group and n = 5 for fMCAo group); differences between groups were determined using two-way ANOVA followed by Holm–Sidak’s test. * *p* < 0.05 and *** *p* < 0.001, fMCAo ipsi vs. sham ipsi, and ^#^ *p* < 0.05, fMCAo contra vs. sham contra; ^++^ *p* < 0.01, fMCAo ipsi vs. fMCAo contra.

There was a substantial global decrease in DCX^+^ cells in the hippocampal DG (Figure 4A,B). The neuroblast numbers decreased in the ipsilateral DG (*p* < 0.0001 vs. sham ipsi) and in the contralateral DG (*p* < 0.0001 vs. sham contra). Within the sham group, the DCX^+^ cell numbers did not differ between hemispheres, and no intra-hemispheric changes were seen in the DG of fMCAo mice, indicating a global decrease in the neuroblast count. The number of mature neurons (NeuN^+^ cells) were not altered in the hippocampal DG of the sham or fMCAo groups (Figure 4A,C). However, when we assessed other hippocampal regions, we observed decreased numbers of NeuN^+^ cells in the hippocampal CA1 region of fMCAo mice (Figure 5A,B). These changes were also global—seen both in the ipsilateral CA1 (*p* < 0.05 vs. sham ipsi) and contralateral CA1 (*p* < 0.05 vs. sham contra). In the hippocampal CA3 region, no significant differences in mature neuron numbers were observed between the groups (Figure 5C,D). Interestingly, no neuroblasts were observed in the contralateral striatum of fMCAo mice, nor in any of the hemispheres in sham mice. The number of NeuN^+^ cells did not differ in the striatum between any of the groups, but were diminished in the ipsilateral cortex of fMCAo mice (*p* < 0.001 vs. sham ipsi, Figure 5E,F). Moreover, this change was hemisphere-specific, as in the contralateral cortex, the number of NeuN^+^ cells was significantly higher (*p* < 0.01 vs. fMCAo ipsi).

### 3.3. Global Changes in Pro-Angiogenic Factor Density after fMCAo

**Figure 6:**
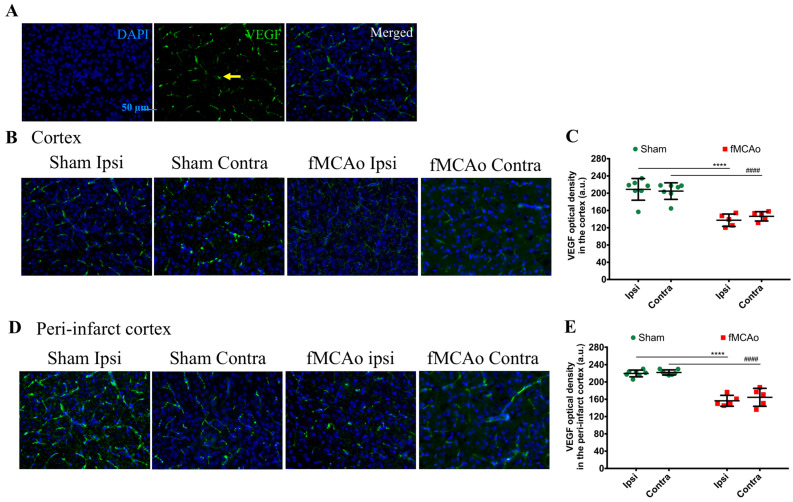
Optical density of VEGF in the ischemia-affected and peri-infarct cortex in fMCAo-group mice and homologous regions in sham mice. (**A**) Representative images of the ischemic cortex/homologous region taken at 20× magnification. VEGF labeling is indicated by yellow arrow. (**B**) Photomicrographs show VEGF staining in the ischemic cortex/homologous region at 20× magnification. (**C**) Graph shows the optical density of VEGF in the ischemic cortex/homologous region. (**D**) Photomicrographs show VEGF staining in the peri-infarct cortex/homologous region at 20× magnification. (**E**) Graph depicting the optical density quantification in the peri-infarct cortex/homologous region. Data are shown as the mean density ± SD (n = 7 for sham group and n = 5 for fMCAo group); differences between groups were determined using two-way ANOVA followed by Holm–Sidak’s test. **** *p* < 0.0001, fMCAo ipsi vs. sham ipsi, and ^####^ *p* < 0.0001, fMCAo contra vs. sham contra.

A global decrease in the optical density of the pro-angiogenic factor VEGF was detected in the ipsilateral cortex of fMCAo mice (*p* < 0.0001 vs. sham ipsi), as well as in the homologous region in the contralateral hemisphere of fMCAo mouse brains (*p* < 0.0001 vs. sham contra, Figure 6B,C). No significant inter-hemispheric differences were observed within the animal groups (sham ipsi vs. sham contra and fMCAo ipsi vs. fMCAo contra).

Moreover, the optical density of VEGF was also markedly lower in the ipsilateral ischemic cortex of the fMCAo mouse brains (*p* < 0.0001 vs. sham ipsi). We observed similar results in the contralateral cortex of the fMCAo-group mouse brains (*p* < 0.0001 vs. sham contra, Figure 6D,E). The VEGF optical density in the homologous cortical region did not differ significantly between the hemispheres within sham controls (sham ipsi vs. sham contra) and fMCAo mice (fMCAo ipsi vs. fMCAo contra).

## 4. Discussion

Increased neurogenesis and angiogenesis are paramount for a successful functional recovery from long-term neurological deficits in an ischemic stroke. Understanding the role of each cerebral hemisphere in these repair processes in the post-stroke brain can improve the design of therapeutic interventions. Our study reports that a 60 min fMCAo induces a global decrease in the neuroblast numbers and pro-angiogenic factor density in both cerebral hemispheres, accompanied by mild atrophy in the ipsilateral hemisphere, after two months.

First, we discovered a dramatic reduction in the neuroblast numbers in the SVZ and hippocampal DG neurogenic niches, suggesting a decreased number of neuroblasts in these regions. In the ipsilateral SVZ of fMCAo mice, the decrease was 77% compared to in the sham controls, whereas in the contralateral SVZ, it was 80%. However, in the hippocampal DG, the deficit was even higher—by 91% in the ipsilateral and 86% in the contralateral DG of the fMCAo mice compared to the sham controls. Previous studies have also reported a sustained decrease in neuroblast production: (1) in the hippocampal DG of both hemispheres in a rat model of cerebral ischemia, albeit at an earlier time point—2 weeks [19]—and (2) in the SVZ of both hemispheres in pericyte-deficient mice 4 weeks after a permanent fMCAo [20]. Both authors used 5-bromo-2′-deoxyuridine-5′ (BrdU) or 5-ethynyl-2′-deoxyuridine (EdU) injections to label the proliferating progenitor cells. Although we did not perform BrdU/EdU injections, the novelty of our report is that we assessed changes in the neuroblast number in both hemispheres of sham animals. In the ischemic brain, neuronal differentiation was not significantly altered in the DG of either hemisphere, similarly to the report by Matsumori et al. [19]. We observed no differences in the formation of new neurons in the hippocampal DG between the sham and fMCAo groups in any of the studied hemispheres. Matsumori et al. also reported that, nine weeks after a 90 min MCAo, the level of neuronal differentiation in the DG was not significantly altered compared to sham-operated controls [19].

Next, we discovered the presence of neuroblasts in the ipsilateral ischemic striatum, a region where these cells do rise in the corresponding area in a healthy brain. Correspondingly, there were no neuroblasts found in the homologous region of the brain of sham-control mice or the contralateral hemisphere of the fMCAo brains. Cerebral ischemia stimulates the migration of adult neural stem cells from the neurogenic niches to the ischemic area [21]. This process occurs subacutely following ischemia and is regulated by multiple transcription factors, as shown in [20]. Previous reports have also demonstrated the presence of neuroblasts in the ipsilateral striatum, earlier—4 weeks after photothrombotic stroke [22]—and later than in our study—for up to 16 weeks after a 120 min fMCAo [23]. In the report by Thored [23], the ipsilateral hemisphere was compared to the contralateral hemisphere, but not to sham controls. Intriguingly, the presence of neuroblasts in the ischemic striatum in our study corresponded a 20% decrease in the number of differentiated neurons in this region. It has been shown that, in the ischemic area, neuroblasts differentiate into astrocytes that limit and rebuild the lesioned area [21]. We suggest that, although neuroblasts were found in the ischemic striatum, their differentiation into functional neurons was limited. In the sham controls (and in the contralateral hemisphere of the fMCAo mouse brains), the numbers of differentiated neurons were significantly higher. In other studies, the death of more than 80% of the differentiated (NeuN+) neurons was reported in the rat ischemic striatum even earlier, at 2–6 weeks after a 2 h fMCAo [23]. Another study showed a similar increase in the neuroblast number in the ipsilateral, but not the contralateral, ischemic striatum 4 weeks after a 120 min MCAo. Notably, no changes in the number of differentiated neurons were observed in the peri-infarct zone in any of the groups. Shibahara et al. reported similar results 4 weeks after a permanent fMCAo in pericyte-deficient mice [20], whereas Matsumori showed decreased numbers of differentiated neurons in the ischemic penumbra 8 weeks after transient ischemia [19]. Our findings are similar and suggest that the migration of the neuronal precursors to the ischemic core does not result in an increased number of differentiated neurons in this region. However, we did not assess the migration of neuronal precursors per se, so this assumption was not proven in this study, nor did we determine whether the neuroblasts present in the ischemia were differentiated into neurons or other cell type, e.g., astrocytes. We can only suggest that, since the neuronal precursors increased in number at the site of the ischemic lesion, the migration took place.

Finally, we observed a deficient availability of the pro-angiogenic factor VEGF in both brain hemispheres of fMCAo mice. VEGF is not purely an angiogenic- and vascular-permeability-increasing factor. Hypoxia after an ischemic injury induces the expression of VEGF that, in turn, promotes neurogenesis and neuronal migration [24]. Hence, VEGF also has neurotrophic and neuroprotective effects. Since neural precursors migrate to the ischemic area along blood vessels and endothelial cell-released VEGF promotes neurogenesis, reduced angiogenesis could contribute to limited neurogenesis and endothelial cell viability in the ischemic area [12]. Our data demonstrate a lowered VEGF density subacutely after an fMCAo. The VEGF density decreased by 36% and 29% in the ipsilateral and contralateral ischemic cortexes of fMCAo mice, respectively. In the peri-infarct region, the VEGF density decreased by 29% and 33% in the fMCAo mouse ipsi- and contralateral hemispheres, accordingly. Our data show that, subacutely after an fMCAo, the VEGF immunoreactivity is 30% lower than physiological levels. Hayashi et al. reported that a 30 min fMCAo produced a gradual decrease in VEGF in the ipsilateral core of mice over 3 weeks [25]. However, the authors did not specifically compare the ipsilateral or contralateral hemispheres between sham and fMCAo animals. In a model of a permanent MCAo, Meng et al. observed an initial increase in VEGF expression in the rat ischemic penumbra for one week, followed by restoration to physiological levels two weeks after the fMCAo [26]. VEGF is reported to enhance hippocampus-dependent memory formation [27], yet we did not observe any changes in VEGF immunoreactivity in the hippocampus. VEGF expression can increase the levels of DCX+ neurons in the hippocampal DG [28]. The decreased levels of immature neurons in the hippocampal DG that we observed in this study may, therefore, be explained by, but not limited to, unaltered VEGF expression. A limitation of our study is that we only determined the VEGF density; to assess the changes in angiogenesis more broadly, one would have to stain blood vessels with lectin [29] or vascular endothelial cells with CD31 [30].

## 5. Conclusions

Data on interhemispheric changes within a particular group (e.g., sham or fMCAo) are scarce in the available literature, and our study serves to partially fill this knowledge gap. Here, we report altered neurogenesis and pro-angiogenic factor density in both hemispheres of fMCAo and sham-group male mice. These data are essential, considering that ischemic changes are believed to take place primarily in the ipsilateral hemisphere. Nevertheless, this is why both hemispheres in both sham and fMCAo animals need to be considered when assessing the changes induced by an ischemic stroke. This is also the novelty of our study—we showed that fMCAo-induced long-term changes do not pertain to the ipsilateral hemisphere only, but also change the neuroblast numbers and VEGF density in the contralateral hemisphere of fMCAo mice. Our data on the recovery processes two months after an fMCAo demonstrate impaired neurogenesis in the stem cell niches and its enhancement in the ischemic striatum. However, a reduced number of differentiated neurons in the ischemic cortex was found, owing to rapid neuronal death due to the ischemic insult. Further studies are required to decipher how an fMCAo chronically affects blood vessel integrity and neural precursor migration, specifically in the contralateral hemisphere. It is possible that the current research will foster interest for assessing long-term observations in both brain hemispheres following an ischemic stroke, and that the effects of novel cardiovascular therapies will be investigated in the whole brain.

## Figures and Tables

**Table 1:** List of the antibodies used in this study.

Antibody	Supplier	Concentration	Cat. No.	RRID
Mouse anti-VEGF	Santa Cruz Biotechnology (Paso Robles, CA, USA)	1:400	sc-53462	AB_630426
Rabbit anti-DCX	Abcam (Cambridge, UK)	1:50	ab207175	AB_2894710
Rabbit anti-NeuN		1:500	ab104225	AB_10711153
Goat anti-mouse IgG (AlexaFluor^®^ 488-conjugated)		1:1000	ab150113	AB_2576208
Goat anti-rabbit IgG (AlexaFluor^®^ 488-conjugated)		1:500	ab150077	AB_2630356
Goat anti-rabbit IgG (AlexaFluor^®^ 594-conjugated)		1:1000	ab150084	AB_2734147

## Data Availability

The data presented in this study are available from the corresponding author upon request. The data are not publicly available due to privacy reasons.

## Supplementary Materials

The following supporting information can be downloaded at: https://www.mdpi.com/article/10.3390/medicina59122168/s1, Figure S1. Microphotographs depicting Nissl staining in rostro-caudal sections of fMCAo group mice brain. Images are shown for slices cut from +2.0 to −2.5 mm relative to bregma (from top left corner to bottom right corner).
