# Supplementary material for: Focal Cerebral Ischemia Induces Global Subacute Changes in the Number of Neuroblasts and Neurons and the Angiogenic Factor Density in Mice
Source: Medicina (Kaunas). 2023 Dec 14;59(12):2168. doi: 10.3390/medicina59122168 (PMC10745011; doi:10.3390/medicina59122168)
Supplement: Supplementary file 1 [file medicina-59-02168-s001.zip › medicina-2750246-supplementary.pdf]

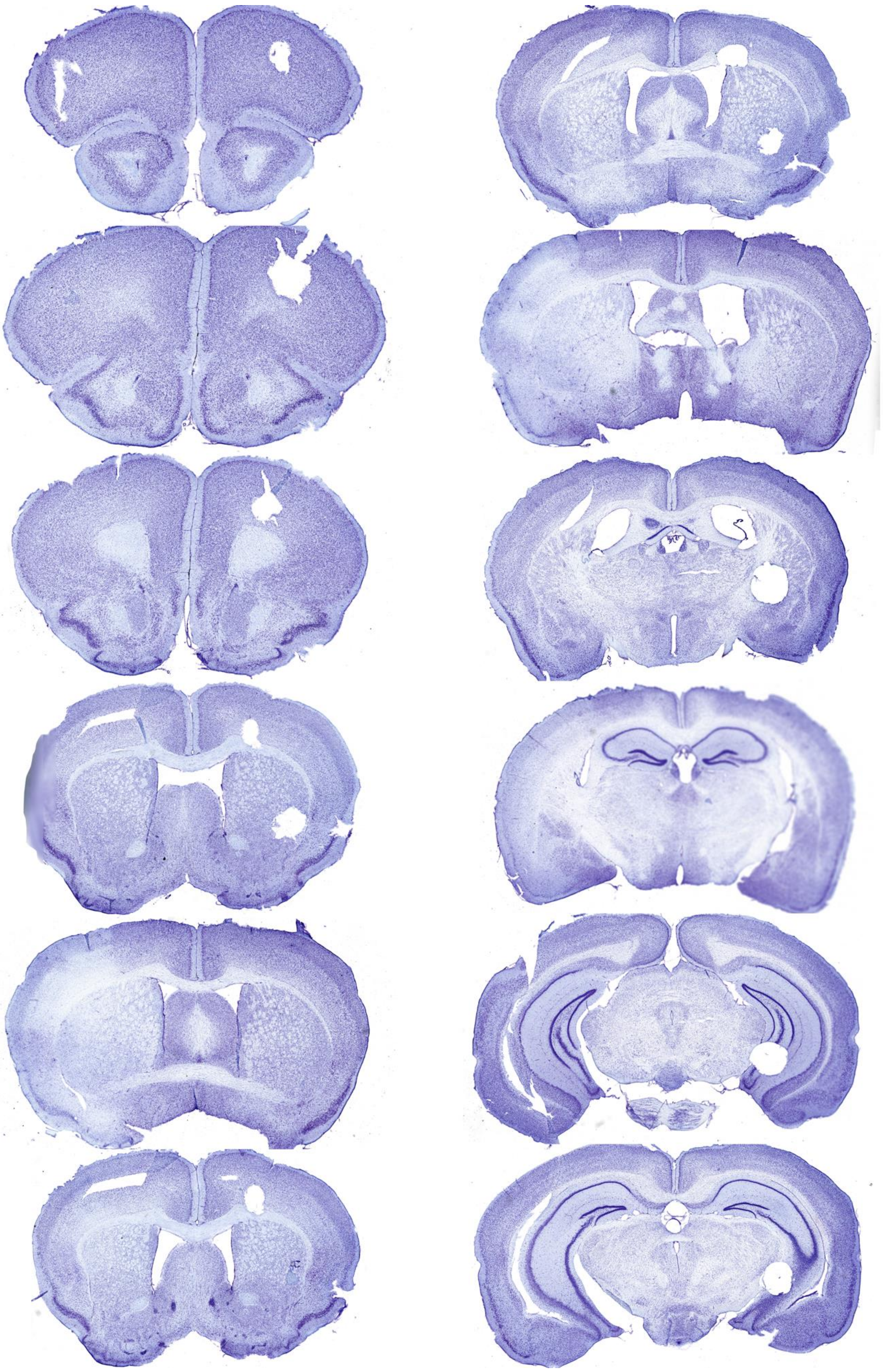

Supplemental Figure S1. **Microphotographs depicting Nissl staining in rostro-caudal sections of fMCAo group mice brain.** Images are shown for slices cut from +2.0 to -2.5 mm relative to bregma (from top left corner to bottom right corner).
